# Prophages in *Bacteroides fragilis*: Distribution and genetic diversity

**DOI:** 10.1016/j.heliyon.2025.e42755

**Published:** 2025-02-18

**Authors:** Paolo Gaibani, Rocco Latorre

**Affiliations:** a Department of Diagnostic and Public Health, Microbiology Section, University of Verona, Verona, Italy; b Azienda Ospedaliera Universitaria Integrata di Verona, Sezione Microbiologia, Verona, Italy; c NYU Pain Research Center, New York University, USA; d Department of Molecular Pathobiology, New York University College of Dentistry, USA

**Keywords:** Prophages, *Bacteroides fragilis* group, Virulence factors, *Fragilysin*, *Environments*, *Pathogenic strains*

## Abstract

**Objectives:**

We evaluated the distribution, epidemiology, and relationships of prophage regions among 500 *Bacteroides fragilis* group genomes.

**Methods:**

Average nucleotide identity (ANI) analysis was carried out to characterize the genome at the species level and phylogenetic analysis was performed to identify the genomic relationship among *B. fragilis* genomes*.* Prophages in B. fragilis genomes were performed with PHASTEST and pairwise comparison of prophage regions was performed by using Jspecies.

**Results:**

Prophages were found in 67.6 % (338/500) of *B. fragilis* genomes with a degree of nucleotide identity >80 % in 54.2 % of these regions. Comparison in the total number of prophages demonstrated that strains belonging to division I showed a higher number of these regions than strains of division II (*p* < 0.01). Characterization of prophage sequences revealed that strains belonging to division I exhibited a lower conservation degree (*i.e.* nucleotide identity ≥90 %) of the nucleotide regions than strains belonging to division II (71.88 % vs 90.0 %; *p* < 0.0001) and strains harbouring toxin-gene showed a lower conservation degree (*i.e.* nucleotide identity ≥70 %) than toxin-negative strains (70.0 % vs 95.4 %; *p* < 0.0001).

**Conclusions:**

We demonstrated a wide distribution and high conservation degree of the prophages among *B. fragilis* genomes. Diversity observed within prophages could reflect the major adaptability of pathogenic strains and that low pressure exerted in the gut of healthy individuals could be related to the high conservation degree of prophage regions in human commensal strains.

## Highlights

• Prophages modulate bacteria population of different anatomical sites.
• Prophages were analysed in 500 *B. fragilis* genomes collected in different countries.
• Prophages were widely distributed in *B. fragilis* with high conservation degree.
• Prophages co-evolved with *B. fragilis* by grouping in the two divisions.
• Prophages were higher conserved in commensal *B. fragilis* genomes.

## 1 Introduction

*Bacteroides fragilis* (BF) is an anaerobic, Gram-negative, rod-shaped bacterium that resides in the human gastrointestinal tract. It is a significant part of the gut microbiota, contributing to the metabolic processes (nutrient absorption and energy metabolism) and maintaining mucosal immunity (immune regulation and intestinal barrier function) [1].

Disturbances in the normal composition of the gut microbiota have been linked to an increased risk of developing diseases. An example is the toxin *Bacteroides fragilis* toxin (*bft*), which has been associated with promoting tumorigenesis by altering cellular signaling pathways and inducing inflammation [2]. Previous studies demonstrated that the presence of bft-producing BF has been found at higher frequencies in the faces of colorectal cancer (CRC) patients compared to healthy individuals, suggesting a possible role in the disease's progression [[2], [3], [4]]. In this context, the genomic diversity of *B. fragilis* strains, particularly those producing bft, underscores the complexity of this bacterium's interactions with human health and disease. Indeed, the genomic diversity of BF genomes contributes to the high adaptability of this microorganism thus reflecting its double role as a commensal bacteria or enteric pathogen [5]. Genetically, *B. fragilis* comprises two distinct groups of clinical treatment importance, named division I and II, which are characterized by the carriage of different antimicrobial resistance genes conferring resistance to Beta-lactams and [6]. In particular, division I carry the *cepA* gene, a serine beta-lactamase, while strains belonging to the division II carry *ccrA* or *cfiA* genes, a metallo Beta-lactamase [6].

In the last years, several studies have demonstrated that bacteriophages (phages) play a key role in the control and distribution of bacteria population and some conditions are involved in the regulation and modulation of several bacteria life processes [7,8]. In particular, genomic and metagenomic analysis has demonstrated that a large proportion of the bacteria genomes carried prophage elements [9] which carried several accessory genes (*e.g.* virulence factors, antibiotic resistance genes, biofilm formation, etc.) that could confer advantages to the bacterial population under specific circumstances [7,10].

Based on these considerations, this study aimed to evaluate the presence of prophages within the *B. fragilis* genomes and define their genomic distribution.

## 2 Material and methods

### 2.1 *Bacteroides fragilis* genome collection, comparative genome and phylogenetic analyses

We collected a total of 500 genomes of *Bacteroides fragilis* acquired from the National Center for Biotechnology Information (NCBI) database *(*https://www.ncbi.nlm.nih.gov/genome/?term=Bacteroidesfragilis*).* The genomes included in this study were selected to delineate the genomic diversity of *B. fragilis group* including strains collected in different geographical area and at different time. For each genome, information on the date and source of isolation, as well as the country and year of isolation, was documented. All data are available in Table S1 in the Supplementary data.

Average nucleotide identity (ANI) analysis was performed to draft or complete genomes included in this study as previously described [11]. Briefly, ANI analysis was performed by comparing each genome to genome of *B. fragilis* NCTC9343 used as reference to identify strain at species level.

Phylogenetic analysis was performed as previously described [12]. The phylogenetic tree based on core genome SNP was generated with Parsnp software with -x -c -a 13 settings using BOB25 (Acc.no. CP011073) as reference genome. Briefly, genomes were aligned to a reference by maximal unique matches (MUMs) to identify the structural and point variations. Phylogenetic tree was rooted at the midpoint and visually using Gingr software.

### 2.2 Prophages analysis

Detection and evaluation of Prophage nucleotide regions among different *B. fragilis* genomes were performed with PHASTEST tools (available online at: http://phastest.ca). The Prophage regions were identified as complete, questionable, or incomplete based on a software algorithm with default settings [13]. Briefly, predicted prophages were identified by comparing the bacterial genomes to phages present in the software database and assigning a score based on the coverage of phages organisms. A score was assigned based on: i) the region only contains genes/proteins of a known phage; (ii) >50 % of the genes/proteins in the region are related to a known phage and (iii) <50 % of the genes/proteins in the region are related to a known phage. For result (i), the region automatically has a completeness score of 150, while for (ii) and (iii) results, the region's completeness score is calculated as the sum of the scores corresponding to the region's size and number of genes. The score obtained was interpreted as follow: intact (score >90), questionable (70–90) and incomplete prophage (<70). The identified Prophage nucleotide regions were used to construct a database for all species included in this study. Calculation of ANI and heat map visualization were performed as previously described [14]. ANI analysis was performed on the Prophage nucleotide regions identified by PHASTEST tool (*i.e.* complete, questionable and/or incomplete) obtained from each genome included in this study. Briefly, pairwise prophage comparison was performed by using JSpecies analysis software with default settings, available online at: https://jspecies.ribohost.com/jspeciesws/. ANI values were calculated for each pairwise comparison and when a Prophage region was compared to itself a score of 100 % was assigned. Heat map visualization was performed using heatmapper software available online at: http://www.heatmapper.ca/pairwise/.

### 2.3 Statistically analysis

Comparison between the number of prophage regions and means was performed using Chi-square and student t-test analysis. All statistical analyses were performed by using GraphPad Prism v.10.1.11 (San Diego, CA, USA).

## 3 Results

**Fig. 1:**
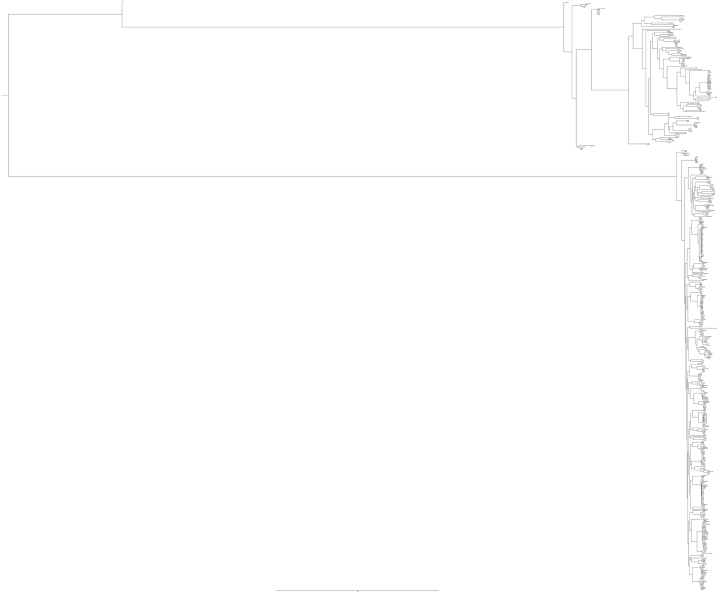
Phylogeny based on the core SNPs analysis of the 500 *Bacteroides fragilis* group genomes included in this study. Phylogenetic tree was rooted at midpoint. BOB25 strain were used as reference genomes.

Overall, the *B. fragilis* genomes included in this study were derived from strains collected between 1955 and 2023 from 19 different countries (Table S1 in the supplementary data). The genomic relationship between genomes demonstrated that all strains belonged to the *B. fragilis* group thus showing an ANI value> 85 % (see Table S2 in the supplementary data). To distinguish between strains belonging to divisions I and II, ANI value was set to a threshold of 95 % as previously reported [11,15]. Our results revealed that 388 genomes belonged to the division I, while 112 belonged to the division II. Similar results were observed by maximum likelihood phylogenetic analysis based on core SNPs, thus showing that 388 and 112 genomes belonged to two different well-supported clades corresponding to the division I and II, respectively (Fig. 1). Analysis of the BTF content showed a statistical difference (*p* < 0.0001) between the two divisions by showing that 22.4 % of the strains belonging to division I, harbored the *bft* gene, while only 2.15 % of the strains belonging to division II were *bft*-positive (data not shown).

**Fig. 2:**
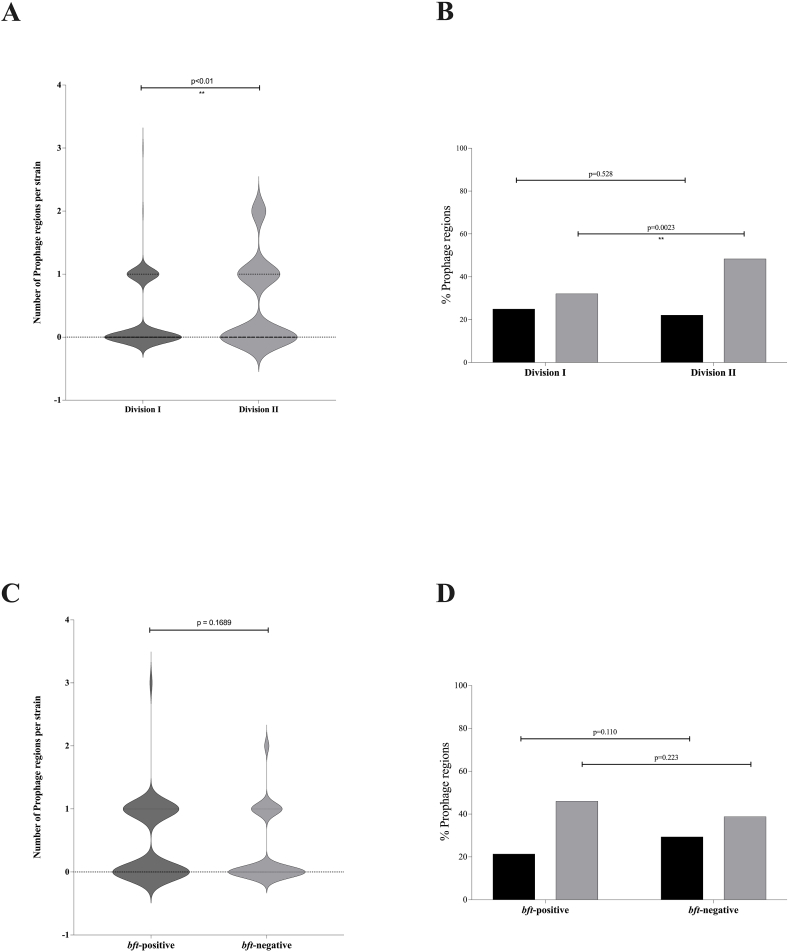
Distribution of prophage regions among *Bacteroides fragilis* genomes. (Panel A) Count of prophage regions per strain among division I and division II. (Panel B) Count of intact or incomplete prophage regions among division I and division II genomes. (panel C) Count of prophage regions per strain among *bft* -positive and -negative strains. (Panel D) Count of intact or incomplete prophage regions among *bft* -positive and -negative strains. Black and grey bars indicate respectively complete and incomplete prophage regions.

To investigate the distribution of prophages within the *B. fragilis* strains, prophage analysis was conducted on the 500 *B. fragilis* genomes included in this study. Overall, 67.6 % (338/500) of the genomes contained prophage regions, while only 25 % (127) of *B. fragilis* strains carried an intact prophage. Analysis of prophages among *B. fragilis* genomes showed that these regions ranging from 9 to 161 Kb (median 41 Kb, IQR 25.5–65 Kb). It is noteworthy that a comparison between two divisions showed a statistically significant difference (p < 0.01) in the total number of prophage regions. This was demonstrated by the fact that 70.61 % (274/388) of the strains in division I and 57.14 % (64/112) of the strains in division II carried prophages. Deep prophage analysis revealed that the total number of the complete prophage regions showed no statistical difference between the two divisions (*p* = 0.528 %), while a comparison of the complete prophage regions per strain revealed statistical differences between the two divisions (Fig. 2, panel A). A comparison of the incomplete prophages revealed that division I had a higher number of these regions compared to division II. (*p* < 0.01) (Fig. 2, panel A).

Comparison in the total number of prophage regions showed no statistical difference between *bft* -positive (68.2 %) and -negative (67.4 %) *B. fragilis* genomes. In addition, no statistical difference in the number of prophages per strain and the number of complete or incomplete regions was observed between *bft* -positive and -negative genomes (Fig. 2, panels C and D). At the same time, no difference between year of isolation and geographical distribution was observed in prophages among the two divisions.

**Fig. 3:**
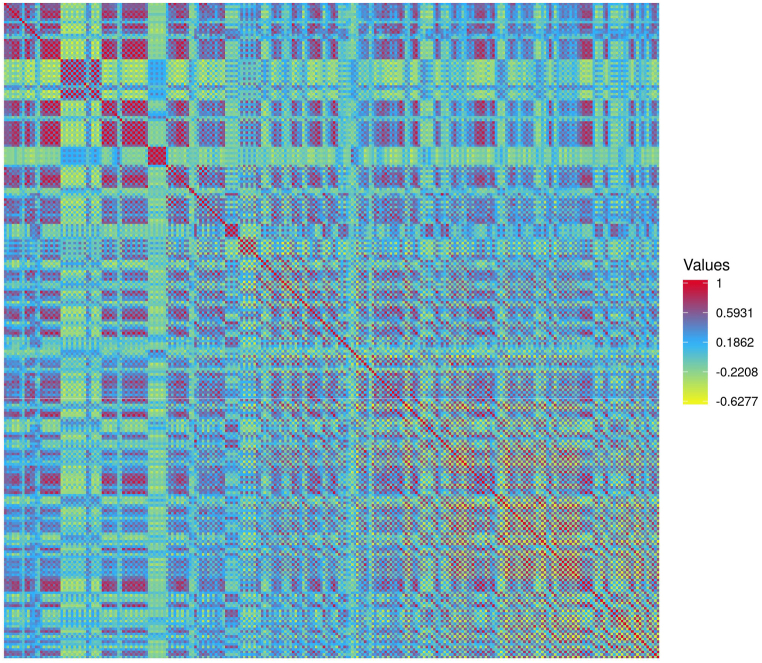
Pairwise comparison among prophages nucleotide regions found among *Bacteroides fragilis* group genomes. The heatmap visualization and clustering were performed by Heatmapper tool and colors represent the ANI values ranging from low (yellow), medium (blue) to high identity (red).

**Fig. 4:**
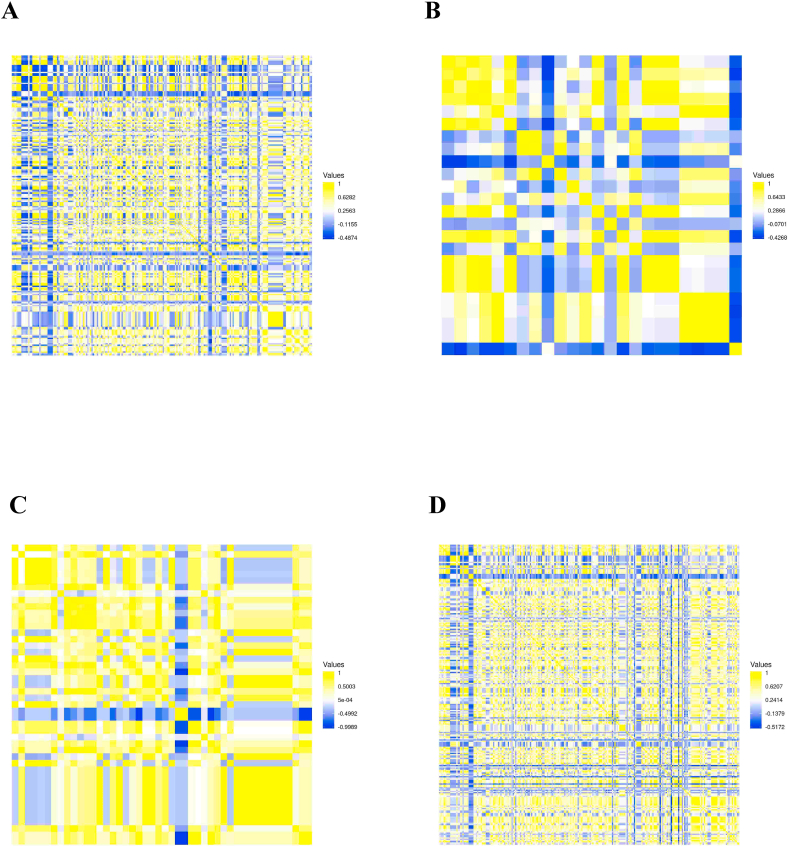
Heatmap of pairwise ANI values in the prophages nucleotide regions among *Bacteroides fragilis* group genomes grouped based on the division and *bft* gene. (Panel A) Prophage regions in the strains belonging to the division I. (Panel B) Prophage regions in the strains belonging to the division II. (Panel C) Heatmap of the prophages regions found in the *bft*-positive strains. (Panel D) Heatmap of the prophages regions found in the *bft*-negative strains. Colors are shown ANI values ranging from low (blue) to high identity (yellow).

To determine the evolutionary relationship among prophage regions within *B. fragilis* strains, sequence homology analysis of the prophage sequences was performed using pairwise comparison. Our results demonstrated that 54.2 % exhibited a sequence homology higher than 80 % among prophages thus demonstrating a high variability in the conservation rate of these nucleotide regions among *B. fragilis* genomes (Fig. 3). Deep prophages analysis revealed a significant statistical difference in the average nucleotide identity between the two divisions by showing that division I exhibited higher variability (*p* *<* 0.0001) on the prophage regions than strains belonging to division II (Fig. S1 in the Supplementary data). In detail, pairwise comparison analysis revealed that strains belonging to division I exhibited a nucleotide identity >90 % in 71.8 % (24119/33590) of the prophage regions, while strains belonging to division II showed a nucleotide identity >90 % in the 90 % (470/552) (Fig. 4 panel A and B). Lastly, pairwise comparison of the prophages regions between *bft*-positive and -negative strains exhibited significant difference (*p* < 0.0001) in the nucleotide sequences homology. In detail, nucleotide sequences homology >70 % were observed in the 70.0 % and 95.4 % of *bft*-positive *bft*-negative strains, respectively (Fig. 4 panel C and D).

## 4 Discussion

In this study, we investigated the presence, distribution, and variability of the prophages in a large collection of *B. fragilis* genomes obtained from strains collected during the last 70 years and distributed in different countries. Our results demonstrated a wide distribution of the prophage regions among *B. fragilis* strains by showing a difference in of prophage numbers and a more conservative similarity in strains belonging to division II and a wide genetic diversity in *B. fragilis* strains of division I. We observed a low conservation degree of the prophages within *B. fragilis* strains harboring the *bfc* gene, with most of them belonging to division I. On the other hand, less variability was observed in the *bft*-negative strains.

It is possible to hypothesize that the integration of prophages within bacterial genomes could play a key role in the variability of pathogenic strains under different stress conditions. Additionally, the high conservation of prophage regions observed within genomes belonging to division II, which contained a low amount of *bft*-positive strains, suggests a relationship with the relatively stable environment within the human intestine of healthy individuals. We can hypothesize that the high adaptation of non-pathogenic strains to the host, being for the majority of them, human commensal in the human intestine, can be associated with the high degree of conservation of their prophage regions especially in species living in restricted habitats such as the gut [6].

These findings are in agreement with previous extensive genomic studies indicating that prophages display a wide range of distribution and complexity among *Lactobacillus* species inhabiting various environments [14]. In this context, the extensive prophages diversity observed in the *B. fragilis* isolates from division I, particularly in strains carrying the enterotoxin gene, may contribute to their adaptation to changing environments during infections in the host's extra-intestinal regions [6].

In summary, our study demonstrated the extensive genetic variability of prophage regions in *B. fragilis* genomes, reflecting the complex and dynamic relationship between phages and microbes. This relationship may play a crucial role in maintaining the balance of human health in infection-related disorders [7]. Recent studies have indicated that imbalances in the gut microbiome may be linked to specific phage compositions, highlighting the significant role of phages in shaping the microbiome [[16], [17], [18], [19], [20], [21]]. In this context, the major genetic diversity observed among prophages regions in *B. fragilis* strains belonging to the division I could reflect a pressure altering the normal homeostasis of a defined body district microbiomes and the ability of *B. fragilis* to adapt to different environments.

## CRediT authorship contribution statement

**Paolo Gaibani:** Conceptualization, Data curation, Methodology, Resources, Software, Supervision, Writing – original draft, Writing – review & editing. **Rocco Latorre:** Resources, Supervision, Validation, Writing – original draft, Writing – review & editing.

## Ethics approval

Not applicable.

## Funding source

This work was supported by FUR 2024 to Paolo Gaibani and MPB Acceleration Fund (P03) to Rocco Latorre.

## Declaration of competing interest

The authors declare the following financial interests/personal relationships which may be considered as potential competing interests:Paolo Gaibani reports financial support was provided by 10.13039/501100007052University of Verona. Rocco Latorre reports financial support was provided by New York University. If there are other authors, they declare that they have no known competing financial interests or personal relationships that could have appeared to influence the work reported in this paper.
