# Supplementary material for: Prophages in Bacteroides fragilis: Distribution and genetic diversity
Source: Heliyon. 2025 Feb 18;11(4):e42755. doi: 10.1016/j.heliyon.2025.e42755 (PMC11891724; doi:10.1016/j.heliyon.2025.e42755)
Supplement: Multimedia component 1 [file mmc1.docx]

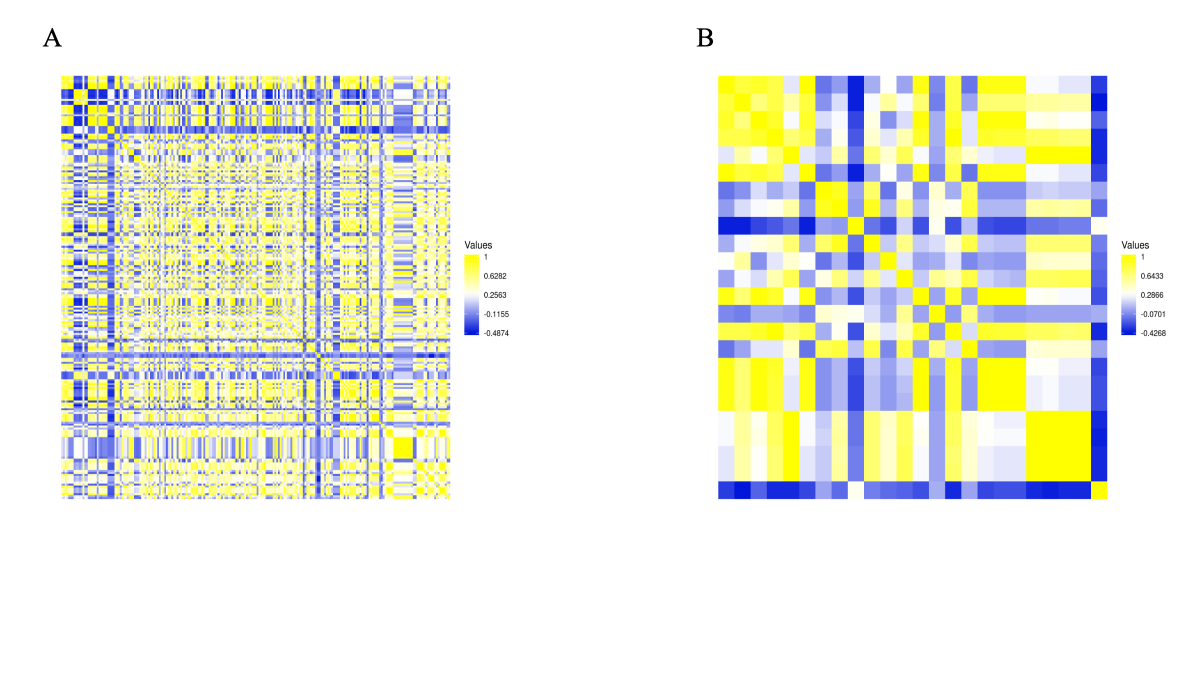
Figure S1. Pairwise comparison among prophages nucleotide regions found among *Bacteroides fragilis* group belonging to the division I (panel A) and division II (Panel B). ANI values ranging from low (yellow), medium (blue) to high identity (red) were shown.
